# Mass-Up: an all-in-one open software application for MALDI-TOF mass spectrometry knowledge discovery

**DOI:** 10.1186/s12859-015-0752-4

**Published:** 2015-10-05

**Authors:** H. López-Fernández, H. M. Santos, J. L. Capelo, F. Fdez-Riverola, D. Glez-Peña, M. Reboiro-Jato

**Affiliations:** Informatics Department, Universidad de Vigo, Campus Universitario As Lagoas s/n, 32004 Ourense, Spain; Instituto de Investigación Biomédica de Vigo (IBIV), Vigo, Pontevedra Spain; BIOSCOPE Research Group, UCIBIO-REQUIMTE, Department of Chemistry, Faculty of Science and Technology, Universidade NOVA de Lisboa, Caparica, Setubal Portugal

**Keywords:** Mass spectrometry, MALDI-TOF-MS, Knowledge discovery, Machine learning, Biomarker discovery

## Abstract

**Background:**

Mass spectrometry is one of the most important techniques in the field of proteomics. MALDI-TOF mass spectrometry has become popular during the last decade due to its high speed and sensitivity for detecting proteins and peptides. MALDI-TOF-MS can be also used in combination with Machine Learning techniques and statistical methods for knowledge discovery. Although there are many software libraries and tools that can be combined for these kind of analysis, there is still a need for all-in-one solutions with graphical user-friendly interfaces and avoiding the need of programming skills.

**Results:**

Mass-Up, an open software multiplatform application for MALDI-TOF-MS knowledge discovery is herein presented. Mass-Up software allows data preprocessing, as well as subsequent analysis including (*i*) biomarker discovery, (*ii*) clustering, (*iii*) biclustering, (*iv*) three-dimensional PCA visualization and (*v*) classification of large sets of spectra data.

**Conclusions:**

Mass-Up brings knowledge discovery within reach of MALDI-TOF-MS researchers. Mass-Up is distributed under license GPLv3 and it is open and free to all users at http://sing.ei.uvigo.es/mass-up.

**Electronic supplementary material:**

The online version of this article (doi:10.1186/s12859-015-0752-4) contains supplementary material, which is available to authorized users.

## Background

Mass spectrometry using matrix assisted laser desorption ionization coupled to time of flight analysers, MALDI-TOF-MS, referred to herein as MALDI, has become popular during the last decade due to its high speed and sensitivity for detecting proteins and peptides. Large sets of samples are analysed quickly in one single batch. The aforementioned reasons have led to the use MALDI for the classification of large sets of samples from different sources and/or characteristics [1]. In this sense, computational tools play a key role in MALDI experiments, as they are able to preprocess raw data registered in different formats, compare them, and apply complex algorithms in order to finally extract new knowledge and useful conclusions.

Raw data generated by MALDI is usually composed of large spectra sets. Each single spectrum contains thousands of measurements entailing mass-to-charge ratio (m/z) signals and intensity (i.e. {m/z, intensity} pairs). These spectra are usually stored using open xml-based formats such as mzXML [2], mzML [3] and PeakML [4]. In addition, several open-source libraries to handle these data formats have been developed in the last years, among which the following are noteworthy: mzMatch [4], jmzML [5], jmzReader [6], the ProteomeCommons.org IO Framework [7] and different R packages [8, 9].

The spectra generated by MALDI apparatus usually contain a high level of noisy signals, making data preprocessing a crucial task that must be carried out before subsequent analysis [10]. This preprocessing is an extensive low-level procedure able to clean raw data and identify true signals in the noisy spectra [11]. Preprocessing comprises several tasks, such as baseline correction, smoothing, normalization, peak detection and peak matching. The use of inadequate or incorrect preprocessing methods can result in a biased dataset, hindering the achievement of meaningful biological conclusions [12]. Therefore, preprocessing is a critical stage in rigorous MALDI data analysis. To accomplish the aforementioned tasks, different algorithms and tools have been developed. Most of them are publicly available as R packages [8, 13, 14], Matlab packages [15], Java libraries [16, 17] or standalone applications [18–20].

Although MALDI is commonly used to identify and characterize molecules, such as peptides or proteins, it can be also used in combination with Machine Learning (ML) techniques and statistical methods [1] to perform biomarker discovery [21, 22], automatic sample classification [23–26], and sample clustering [27, 28]. However, there are no tools devoted to performing these analyses, thus forcing researchers to use more general tools such as R, SPSS, Weka [29] or RapidMiner [30] to carry out them. This makes it necessary to include an intermediate adaptation step to convert the preprocessed MALDI data into the input format required by each tool.

In order to make the development of mass spectrometry (MS) proteomics applications easier, some frameworks such as OpenMS [31] and ProteoWizard [32], in C++, and MsInspect [16] in Java have been published. An example of a tool developed using such frameworks is TOPP (The OpenMS Proteomics Pipeline) [33], which is based on the OpenMS framework.

In spite of the existence of such a great variety of tools and techniques for both the preprocessing and data analysis of MALDI based proteomic datasets, there is still a lack of specific tools that cover the whole process of MALDI data analysis, allowing the users to manage raw datasets, preprocess them and perform several analyses in a row, and allow the user to apply different ML and statistical techniques to analyze MALDI data. Moreover, most of the tools are intended to be used by a user with a bioinformatic profile, requiring programming skills.

This paper presents Mass-Up, an extensible open-source platform for MALDI data processing and analysis with ML and statistical techniques that has arisen from our previous experience working with MALDI data [34–36]. Mass-Up is an AIBench [35] based desktop application specifically created to perform complete analyses of MALDI data, allowing the users to: (*i*) import raw data from different formats (mzML, mzXML, csv); (*ii*) preprocess raw data; and (*iii*) perform different type of analyses, including supervised (e.g. biomarker discovery, predictor building, etc.) as well as unsupervised (e.g. clustering, biclustering, etc.) techniques.

The Mass-Up design is focused on two main objectives: coverage of the whole process of data analysis and simplicity of use. The first objective is accomplished in the way Mass-Up covers the whole process of MALDI data analysis, from data preprocessing to different types of analysis. The second is achieved through a design that allows Mass-Up to be used in a straightforward manner by non-informatician users. In addition, Mass-Up is multiplatform, open source and designed using a pluggable architecture which makes it easier for programmers to develop and include new algorithms and analysis tools.

## Implementation

Mass-Up is a computer application for managing, preprocessing and analyzing MALDI data. Mass-Up is implemented in Java and it was constructed using the AIBench framework, which has been demonstrated to be suitable for developing proteomics applications [36], as it is the base framework of previously developed MS applications [37, 38]. Currently, Mass-Up has distributions for Windows and Linux operative systems.

This section briefly describes the Mass-Up workflow and the main algorithms and third-party libraries employed in each Mass-Up task.

### Mass-Up workflow

**Fig. 1:**
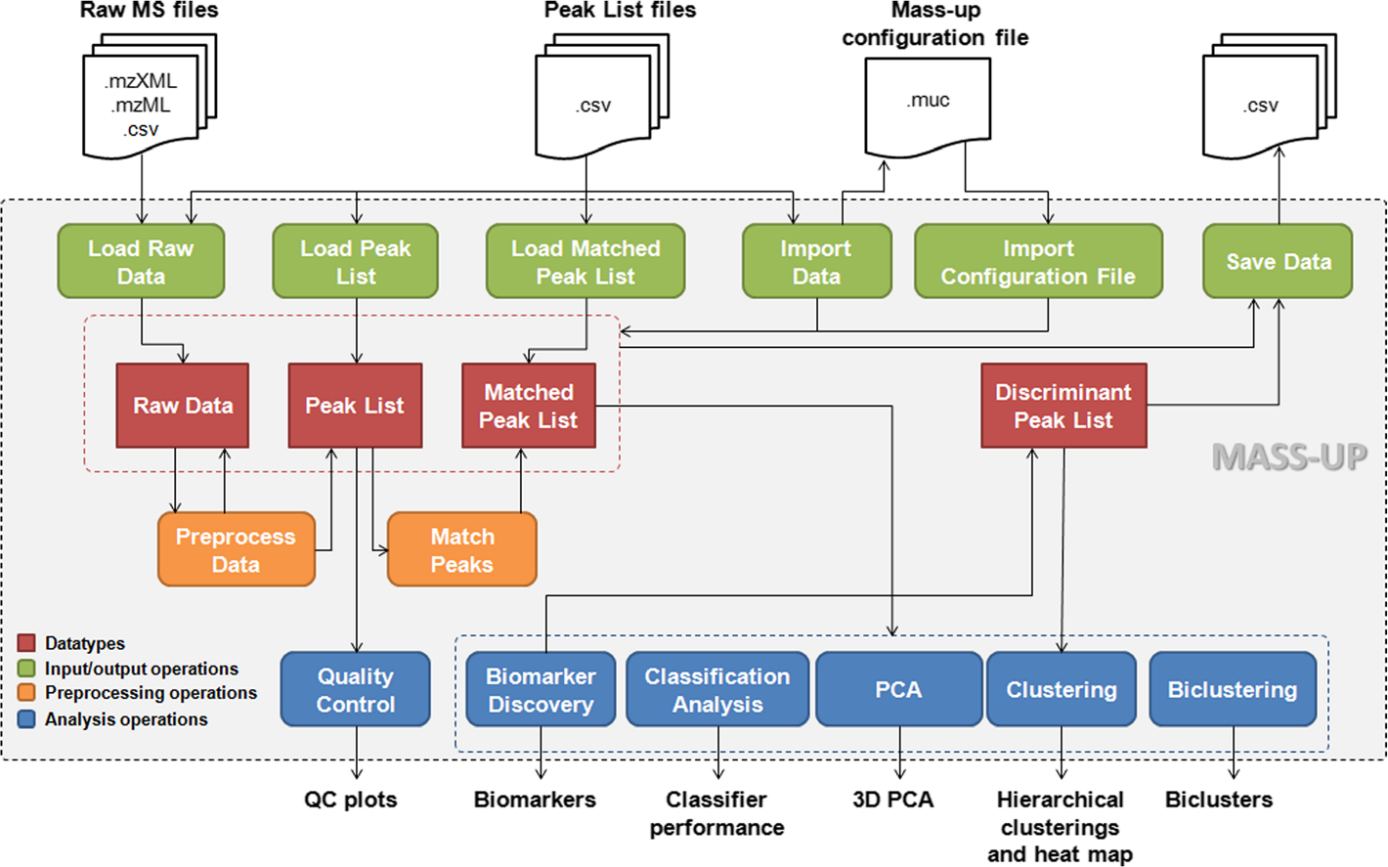
Mass-Up main workflow. Mass-Up main workflow operations and datatypes. Different colors have been used to identify input/output operations (green), preprocessing operations (orange), analysis operations (blue), and datatypes (red)

Mass-Up includes a serie of operations that can be classified into (*i*) input/output operations, (*ii*) preprocessing operations, and (*iii*) analysis operations. Figure 1 depicts the Mass-Up main workflow, where the most important operations are represented, along with the input files and data types managed by the application.

### Third-party libraries

With the main goal of covering the whole process of MALDI data analysis, Mass-Up integrates several open source third-party libraries in order to accomplish different tasks, such as reading different MS data formats, preprocessing spectra, applying ML techniques, or visualizing data, among others. Additional file 1: Table S1 shows a general overview of the Mass-Up, including the algorithms and libraries used by each operation. All of these libraries has been transparently integrated into Mass-Up so that final users does not have to install them manually, since they are built-in in each Mass-Up distribution.

Mass-Up uses jmzReader 1.2.0 [6] in order to read the mzXML and mzML MS data formats. To visualize MS spectra and to display quality control charts, Mass-Up uses JFreeChart 1.0.13, an open source Java library.

Mass-Up integrates two R packages for raw MALDI data preprocessing: MALDIquant [8] and MassSpecWavelet [13]. In addition, custom implementation of a fast peak matching algorithm based on a forward sliding window, named *Forward*, is also incorporated. Similarly to the alignment algorithm proposed by Kazmi et al. [39], this algorithm iterates the peaks from minimum to maximum m/z, adding them to the last cluster created if their m/z is within a distance from the average m/z of the cluster or creating a new cluster if not. This clustering algorithm does not allows clusters with two peaks from the same spectrum. In such case, only the peak that minimizes the average m/z of the cluster is kept.

Mass-Up makes use of Weka [29], a collection of ML algorithms for data mining tasks implemented in Java. These algorithms are used for classification and for principal component analysis (PCA). Three-dimensional PCAs are rendered by using Jzy3d [40], an open source Java library which can easily draw three dimensional scientific data. Clustering is executed using a custom implementation of an agglomerative hierarchical clustering algorithm and is rendered using an adapted version of JTreeView [41].

Biclustering is performed with Bimax [42], a powerful algorithm capable of generating all optimal biclusters, and BiBit [43], a novel approach for the extraction of biclusters from binary datasets that can obtain similar results to Bimax by using significantly less computation time and reducing the total number of generated biclusters. The aforementioned software, as well as a biclusters viewer, is integrated through the adaptation available in BiMS [44].

## Results and discussion

Mass-Up is a flexible tool that includes several operations whose application depends on the analysis objectives. Therefore, there is no single way to use Mass-Up, and researchers must determine which analyses apply in their studies. In this section, several practical applications of the Mass-Up operations are presented, in order to demonstrate its usefulness and applicability.

### Sample datasets

Two datasets from previous studies were selected to illustrate the Mass-Up functionality. A brief description of the main characteristics of both datasets is given in this section.

#### Cancer dataset

R. López-Cortés *et al*. [45] propose the use of gold-nanoparticles to separate the proteins and peptides in human serum as a way to improve MALDI-based sample profiling. The protocol described in this work divides each sample into two sub-samples: pellet and supernatant. The MALDI spectra of both sub-samples are grouped by their corresponding conditions using three-dimensional PCA. The dataset is composed of sera from 5 patients with lymphoma, sera from 5 patients with myeloma, and sera from 2 healthy donors. As the clasifications using pellet or supernatant are similar, only the latter sub-samples are used in the present work.

#### Wine dataset

Nunes *et al*. [46] propose a fast MALDI-based methodology to identify different types of wines. The authors carry out a preliminary study with 5 wines of different denominations of origin, in order to identify the most appropriate MALDI matrix. The study of the matrices found that CHCA is the most suitable for the purpose of classification. Each wine was spotted five times (i.e. 25 samples in total). Those 25 samples corresponding to the use of CHCA matrix are used as proof of concept.

### Preprocessing

As previously stated, the preprocessing of MS data is a critical stage that converts raw data into a suitable input for further analysis. Inadequate or incorrect preprocessing methods can result in biased datasets, hindering the achievement of meaningful biological conclusions [12]. Preprocessing is essential since raw data contains both m/z values belonging to analytes, as well as m/z values derived from several forms of noise (e.g. chemical, electronic factors, etc.). The main objectives of preprocessing are [47] to remove noise without discarding any of the m/z values of interest, and to determine the m/z and intensity values with the best accuracy. The most common preprocessing tasks (shown in orange in Fig. 1) are smoothing, baseline correction, normalization, peak detection, and peak matching.

Usually, spectra are jagged, making it difficult to detect the m/z values of interest from the noise [47]. Thus, smoothing algorithms are usually applied to soften the spectra. The simplest techniques are based on the use of a sliding window, where the intensity of each m/z value is adjusted based on the intensity of the neighbor m/z values. Commonly used filters are moving average, Savitzky-Golay, Gaussian and the Kaiser window. Mass-Up provides two smoothing methods: moving average window and Savitzky-Golay, both from the MALDIquant library [8].

Baseline is a specific form of noise mainly driven by chemical perturbations, defined as an offset of the intensities of peaks that often show a dependency on the m/z value such that it is highest at low m/z values, presenting an exponential decay towards higher masses [47]. The most common baseline correction methods are monotone minimum, linear interpolation, LOESS, moving average of minima and continuous wavelet transform, all of which are available as free software in different packages such as Cromwell [15] (Matlab), PROcess [14] (R), MALDIquant [8] (R) or SpecAlign [19] (Java). Mass-Up allows the user to make use of all the baseline correction methods provided by MALDIquant (i.e. Top Hat, SNIP, Convex Hull, and Median).

A major constraint of MALDI is that the intensity of the m/z values is relative and can vary among spots of the same sample. For this reason, normalization is typically used, making the intensities of different spectra comparable. The most common normalization methods are Total Ion Current (TIC), Probabilistic Quotient Normalization (PQN), Z-score, Linear, Mean or Median. Mass-Up allows the user to perform normalization using TIC, PQN or Median, all provided by the MALDIquant library [8].

The m/z detection can be defined as the process of selecting values of interest (i.e. related with target analytes) from a given spectrum, and it is normally applied after baseline correction and smoothing. Most of the peak detection methods are based on setting a threshold value in order to discard low intensity m/z values. The threshold can be absolute (e.g. minimum intensity) or relative (e.g. signal-to-noise ratio, SNR). However, Du *et al*. [13] proposed a method that performs m/z detection without explicit smoothing and baseline correction. This method is based on the continuous wavelet transform (CWT) and is publicly available in the MassSpecWavelet package. Mass-Up includes two m/z selection methods: the CWT-based method implemented in MassSpecWavelet [13], and a SNR-based method provided by MALDIquant [8], which uses a sliding window.

Finally, m/z matching is needed in order to make different spectra comparable. Without this matching procedure, the same molecule or metabolite (e.g. a certain peptide) can have different m/z values across replicates or samples. The objective of m/z matching methods is to find a common set of m/z locations in several spectra, so that all spectra will have the same m/z values for the same biological entities. In Mass-Up there are two fundamental types of m/z matching: intra-sample and inter-sample. The intra-sample matching is applied to the spectra obtained for the replicates of the same sample, while the inter-sample matching is applied to match m/z values across different samples, making them comparable and suitable for the subsequent analysis stage. Peak matching algorithms, are classified into two main groups: sequential algorithms based on a sliding window (e.g. the *Forward* algorithm, available in Mass-Up) and clustering based approaches (e.g. the MALDIquant algorithm [8], also available in Mass-Up).

The Mass-Up workflow also incorporates an additional filtering step that is very closely related to the matching process. This step is performed after the intra-sample matching and before the inter-sample matching, and allows the creation of a consensus spectrum for a sample, which summarizes the replicates of a sample in one single spectrum. In this step, the Percentage of Presence (POP) parameter allows the user to set the number of replicates where an m/z value must be present in order to be considered a valid consensus m/z value.

Finally, it is important to note that, while smoothing, baseline correction, normalization, and m/z detection are applied individually to each single spectrum in the *Preprocess data* operation, the m/z matching is applied to several spectra at the same time and is carried out by using the *Match Peaks* operation.

The new data generated by the *Preprocess data* and *Match Peaks* operations can be exported as comma-separated value files, allowing users to load them later with Mass-Up or to analyse them with other software packages. Mass-Up documentation includes information about exporting data and examples describing how it can be loaded in other languages such as R.

### Quality control

When working with MALDI, low quality spectra may occasionally be generated. For example, spectra showing a low number of m/z values in comparison with other spectra, or containing many unique m/z values not present in their sibling replicates. These spectra may lead to failure when carrying out an analysis, or to incorrect conclusions. To prevent such a scenario, a quality control (QC) step was included, which may be performed between the preprocessing and the analysis tasks. The QC can be done at two levels: *replicates*, a low level QC analysis focused on the replicates of each sample; and *samples*, a high level QC analysis with additional information from the intra-sample m/z matching process.

**Fig. 2:**
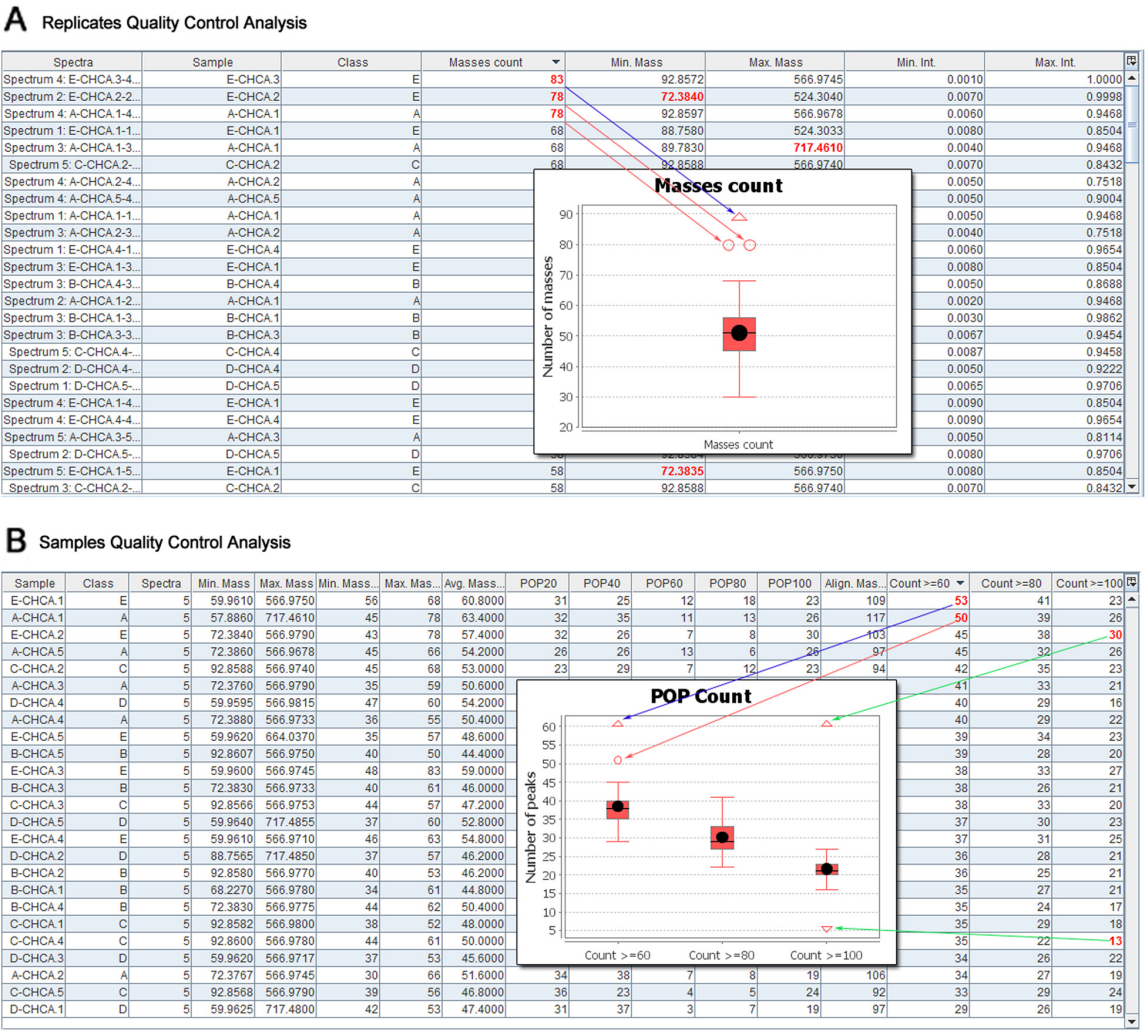
Quality control view. Details of the quality control analysis views for **a** replicates, and **b** samples. Box plot charts are used to summarize the more detailed information presented in the tables

At the replicates level, the user can check basic information about each individual spectrum (i.e. peak count, m/z range, intensity ranges, etc.) and compare all spectra in the dataset. Figure 2a shows a replicate QC analysis applied to the samples from conditions A, B, C, D, and E of the Wine dataset previously described. As can be noted from the boxplot, there are two outliers (red circles) and one extreme outlier (red triangle) in the Masses count chart. Specifically, the QC analysis has marked the E-CHCA.3-4, A-CHCA.1-4 and E-CHCA.2-2 samples as outliers due to the number of m/z values of their spectra. Therefore, before continuing with further analysis, it is recommended to carefully revise these samples and even to repeat their analysis.

At the samples level, the user can check the performance of the intra-sample peak matching process, by comparing the percentages of presence (POP) counts (globally and by conditions) and the POPs of each sample. As previously stated, the data table is more detailed and contains additional information from the intra-sample peak matching process, specifically: (*i*) POPXX columns, where XX is a percentage of the number of spectra, which show the number of peaks with a POP value exactly equal to XX; (*ii*) Align. Masses column, which shows the number of masses that have been matched across the spectra in the sample; (*iii*) Split > = XX columns, which show the percentage of masses that have a POP value higher or equal to XX; and (*iv*) Count > = XX columns, which show the number of masses that have a POP value higher or equal to XX (these are the columns used as categories in the charts).

Figure 2b shows a samples QC applied to the same samples as in the previous example. In this case, the box plot corresponds to the global POP count and shows that there are two outliers for the category “Count > = 60” and one outlier for the category “Count > = 100”. Again, the outliers are highlighted in bold in the table.

### Biomarker discovery

One of the main purposes of the MS analyses is the biomarker discovery [21, 22, 48]. A biomarker is a peptide, protein or other element of a sample that can identify and differentiate certain conditions such as phenotypes, strains, diseases or infections.

When identifying new biomarkers, it is necessary to distinguish between two types of data sets that can be analyzed: (*i*) those cases where there are a known and well defined number of conditions (e.g. healthy vs. diseased, differents stages of a disease, etc.), and (*ii*) those cases where there are no conditions or where they are not clearly defined. In accordance with this differentiation, Mass-Up provides two types of biomarker discovery analysis: (*i*) the inter-label analysis, for the former type of data, and (*ii*) the intra-label analysis, for the latter.

In the inter-label analysis, the user can perform the appropriate statistic tests to identify those peaks that can be potential biomarkers to differentiate the conditions. Four different tests of independence were included in Mass-Up following the recommendations given by McDonald [49], where tests are chosen depending on the number of samples and conditions of the dataset, as shown in Table 1. Taking into account that the number of samples in MALDI experiments is generally below 1000, the Fisher’s exact test and the randomization test are the tests more commonly applied. As each test is performed independently for each m/z value, the Benjamini-Hochberg FDR correction is applied to take into account the number of m/z values analyzed and reduce the number of false positives.

**Table 1:** Tests of independence applied depending on the number of samples and conditions

	<= 1000 samples	>1000 samples
2 conditions	Fisher’s exact test	Yates’ chi-square test
>2 conditions	Randomization test	Chi-square test

**Fig. 3:**
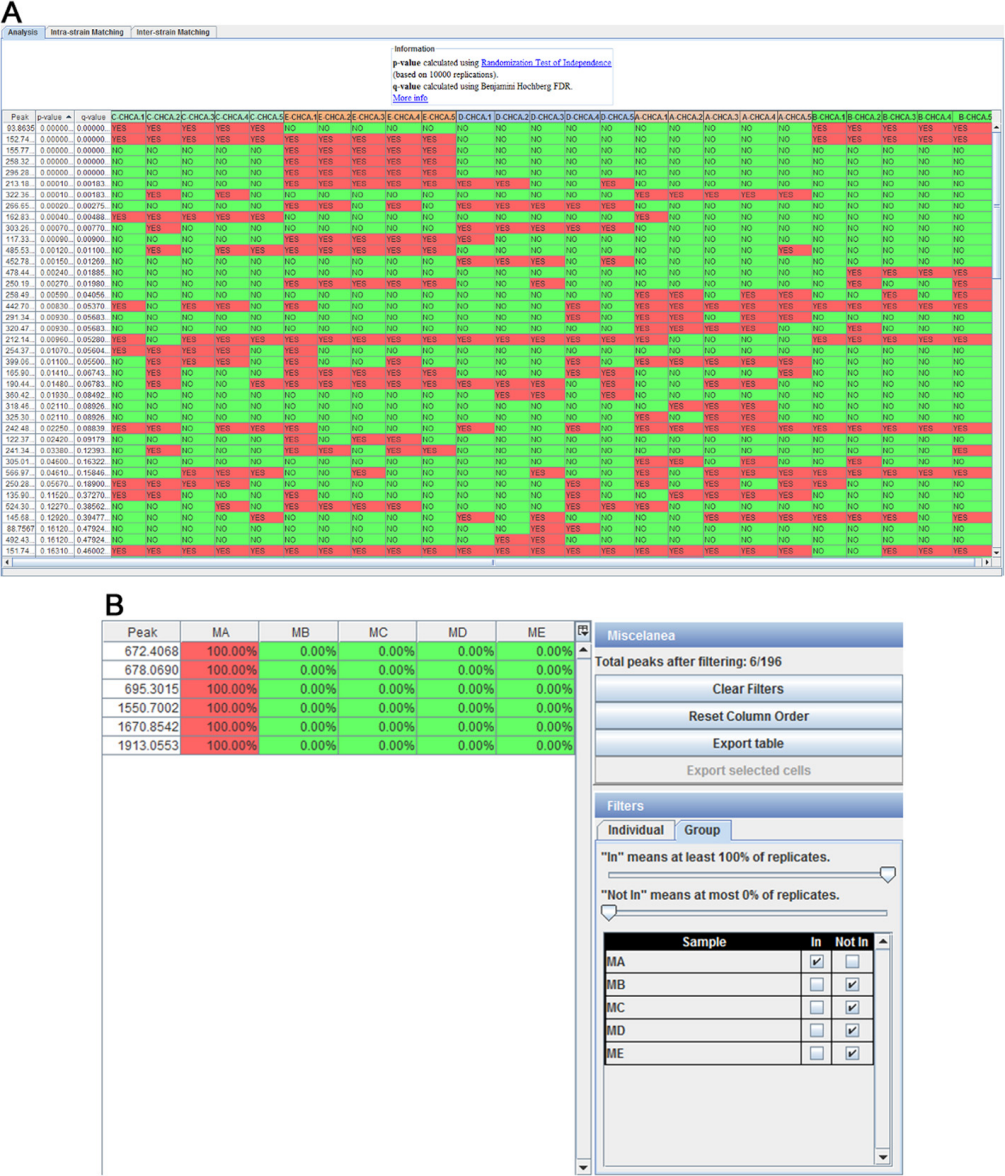
Inter-label and intra-label biomarker discovery analysis views. **a** Inter-label biomarker discovery view. Depending on the number of samples and conditions, Mass-Up automatically selects the appropriate statistical test to apply. **b** Intra-label biomarker discovery view. Filters are configured to select only the m/z values present in the MA samples and absent in the other samples

By using the inter-label analysis in the Wine dataset (shown in Fig. 3a), we can analyze all the samples of conditions A, B, C, D, and E. In this case, the randomization test is applied in order to identify statistically relevant m/z values, as the number of samples is lower than 1000 (5 samples for each of the 5 wines for a total of 25 samples) and the number of conditions is higher than 2 (5 wine denominations). The first three columns contain the m/z value, the p-value, and the q-value respectively; while the other columns show in which samples the m/z values are present. As can be seen, the peaks with a q-value < 0.05 are clear candidates to be biomarkers as they differentiate certain conditions from others.

In the intra-label analysis, the user can identify those m/z values that are representative of one or more samples, in a more exploratory fashion. In this scenario, it is possible to identify the biomarkers of a specific sample or discover groups of samples with a similar profile that may, therefore, be related. This analysis is particularly useful, for example, when working with different strains of the same bacteria and the user wants to identify those peaks that are unique for a certain strain.

By using the intra-label analysis in the Cancer dataset (shown Fig. 3b), we can analyze the samples of the condition Myeloma and configure the analysis to identify those peaks present in the “MA” sample (i.e. Myeloma A) and not present in the rest of the samples. The identification of these peaks may be useful, for example, to explain the abnormal behaviour of a sample when compared to other samples from the same condition. Specifically, the Intra-label Biomarker Discovery view shows how we are looking for specific peaks of the sample MA (i.e. Myeloma A), that is, peaks that are in this sample but not in the others.

### Principal component analysis

PCA is a mathematical procedure that uses orthogonal transformation to convert a set of observations (i.e. samples) of possibly correlated variables (i.e. m/z values) into a set of values of linearly uncorrelated variables called principal components (PC), whose dimensionality is expected to be lower than the dimensionality of the original data set.

Once the PC are calculated, they can be used to represent the samples in a 3-dimensional space. By assigning a different color to each condition’s samples, users can visually identify if there is a separation between conditions. If such were the case, then the conditions would be distinguishable. The PCA view also includes additional information about the PCA, such as the eigenvectors and their corresponding eigenvalues and retained variances, for a better results interpretation.

**Fig. 4:**
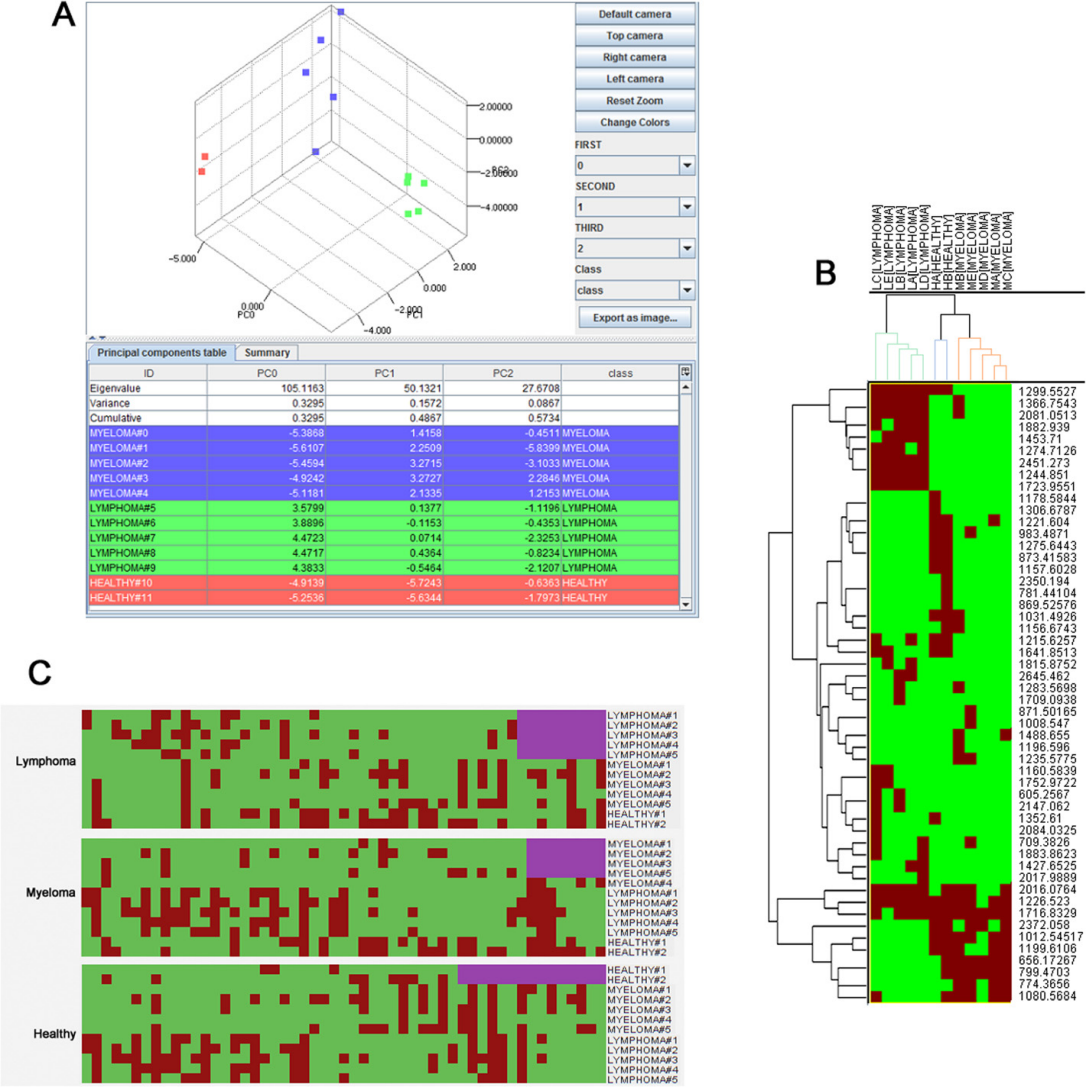
PCA, clustering, and bi-clustering analysis views. **a** Principal component analysis view presenting three different clusters, one for each condition. **b** Detail of the hierarchical clustering visualization using JTreeView. The upper dendrogram automatically colors the tree branches that only include samples from the same condition, while the side dendrogram groups the more similar m/z values. **c** Class-biclusters of the Cancer dataset extracted with the Mass-Up Biclustering Viewer. Purple rectangles denote the existence of biclusters associated with one condition

As previously stated, López-Cortés *et al*. [45] demonstrate that the spectra of supernatant sub-samples of the Cancer dataset can be grouped by their corresponding conditions using PCA. Figure 4a shows the result of applying PCA to this set of samples in Mass-Up. As it can be clearly seen, the three conditions are separable in the 3-dimensional space.

### Cluster analysis

Cluster analysis allows finding groups of similar spectra among all the samples being studied. In the case of unlabeled data, it allows discovering hidden or previously unknown subgroups of samples. In the case of labeled data, it allows the user to check if the different conditions present in a dataset are separable by means of the m/z values of each sample.

Mass-Up incorporates a hierarchical clustering algorithm for the construction of a hierarchy of sample groups (named clusters). The algorithm included is agglomerative and follows a bottom-up approach, meaning that it is constructed iteratively, starting with each sample in its own cluster, and merging the closest pair of clusters on each step. In order to decide which clusters should be merged, a measure of dissimilarity between clusters is required. In our case, this is achieved by using a distance metric, which measures the distance between two samples, and a linkage criterion, which specifies the dissimilarity of clusters. Mass-Up includes the Euclidean and Hamming distances as distance metrics, and the complete, single and average functions as linkage criteria. The results of a hierarchical clustering are usually presented in a dendrogram.

An important aspect when performing a cluster analysis in Mass-Up is that the user can decide whether to use intensities (i.e. a m/z value is represented by the value of its peak intensity) or not (i.e. a m/z value is represented by its peak presence or absence). The Euclidean distance is the most suitable when using intensities while the Hamming distance is the most appropriate when using presence/absence of peaks.

In each cluster analysis, two hierarchical clusterings are constructed: one for the samples and one for the m/z values. For the visualization of the results, Mass-Up incorporates an adapted version of JTreeView, a software for the visualization and analysis of gene expression data. We have adapted it to MS, so that in our specific case the rows represent peaks instead of genes, while columns still represent samples. This representation also includes a heat map, which is combined with two dendrograms that represent the aforementioned hierarchical clusterings. The individual values contained in the heat map matrix are displayed as colors and they can represent (*i*) the intensity level of the corresponding peak (red if the peak has an intensity value of 1; green if the peak has an intensity of 0; and intermediate colors for intensities between 0 and 1), or (*ii*) the presence or absence of the peak (red if the peak is present and green if the peak is not present). It is important to note that to achieve a correct representation using intensities, the m/z values must be scaled between 0 and 1 during the raw data preprocessing.

Figure 4b shows the results of applying hierarchical clustering to the Cancer dataset used as proof-of-concept. As the dendrogram illustrates, the three conditions are well separated since the samples of each condition can be grouped together.

Finally, it is worth noting that the cluster analysis can be used with a list of previously selected peaks. This way, the cluster analysis will be focused on analyzing only these peaks. This list can be obtained by exporting the biomarkers identified in the inter-label analysis. In such a situation, this feature is useful to qualitatively verify if a list of potential biomarkers is enough to separate or differentiate between the conditions of study.

### Bicluster analysis

Although biclustering techniques have been successfully used with gene expression data for over a decade, it is only very recently that those techniques have been applied to MS data [50]. Biclustering is a data mining technique that allows simultaneous clustering of the rows and columns of a matrix. It has been successfully applied to analyze microarray data due to their ability to discover co-expressed genes under certain samples [51]. In contrast to traditional clustering techniques, where each gene in a given cluster is defined under all the samples, biclustering algorithms propose groups of genes that show similar activity patterns under a subset of the experimental samples.

In previous studies, we have proposed a novel workflow for the application of biclustering to MALDI data. In addition, the adequacy of applying biclustering to analyze such data by comparing biclustering and hierarchical clustering over two real datasets has also been evaluated [44]. Biclustering has shown the ability to discover groups of samples that are similar but only in a subset of m/z values, which represent a new kind of hidden hypothesis that are difficult to be discovered by classic clustering algorithms, such as hierarchical clustering, which are based on a global comparison of samples including all m/z values.

The biclustering algorithms selected in the study and included in Mass-Up (i.e. Bimax and BiBit) use a binary dataset as input where 1 represents a peak presence, and 0 represents a peak absence. These algorithms will look for groups (i.e. biclusters) of 1’s, that we call presence patterns. Nevertheless, in certain cases, it can be desirable to extract other type of patterns, such as absence patterns (i.e. biclusters of 0’s) or simple presence/absence patterns (i.e. biclusters of 1’s and 0’s in one direction). López-Fernández *et al*. [44] further discuss how to prepare an input MALDI dataset into a suitable form to look for these three types of patterns.

Mass-Up provides an operation to apply this technique to both labeled and unlabeled samples. The user has to select the biclustering algorithm to use, the type of pattern and the biclustering mode (i.e. whether rows of the biclustering binary matrix are peaks or samples). In addition, the user can also establish the minimum dimensions of the output biclusters. If the input data is labeled, the user can also indicate whether the output of the biclustering must be filtered in order to only retrieve those biclusters where most of the samples belong to the same condition or label, known as class-bliclusters. After performing a biclustering analysis, results can be inspected in the biclustering viewer, an intuitive view that shows a list of the generated biclusters as well as a heat map. If a bicluster is selected, it will be highlighted in the heat map, which is automatically rearranged in order to show the bicluster in the upper left corner.

In order to demonstrate the usefulness of this module, we considered the Cancer dataset used in previous sections, and applied biclustering by means of the BiBit algorithm in the hope of finding presence class-biclusters. Figure 4c shows one presence class-bicluster for each class, where each column represents one m/z value and each row represents a sample. As shown, each class bicluster includes a group of m/z values with the same pattern of presence in the samples of one condition, and a variable pattern of presence in the rest of the samples. When using a presence class-bicluster, only presence is taken into account to create the class-bicluster, whereas when using a presence/absence class-bicluster, the absence is also taken into account.

### Classification analysis

Sample classification is the ability to predict the label of a sample given a training set of labelled samples, therefore, the capacity of producing a diagnosis machine [10, 24, 26]. Through the “Classification Analysis” operation, the user can determine which classifier performs best for the data under analysis. This operation provides an interface adapted from the Weka software that allows the user to select and to configure a classifier, and to evaluate its performance by means of a cross-validation scheme. The output log of the evaluation process summarizes the performance of the classifier using different statistical measurements, such as accuracy, kappa, precision, recall, etc. In addition, you can make a receiver operating characteristic (ROC) analysis per condition.

**Fig. 5:**
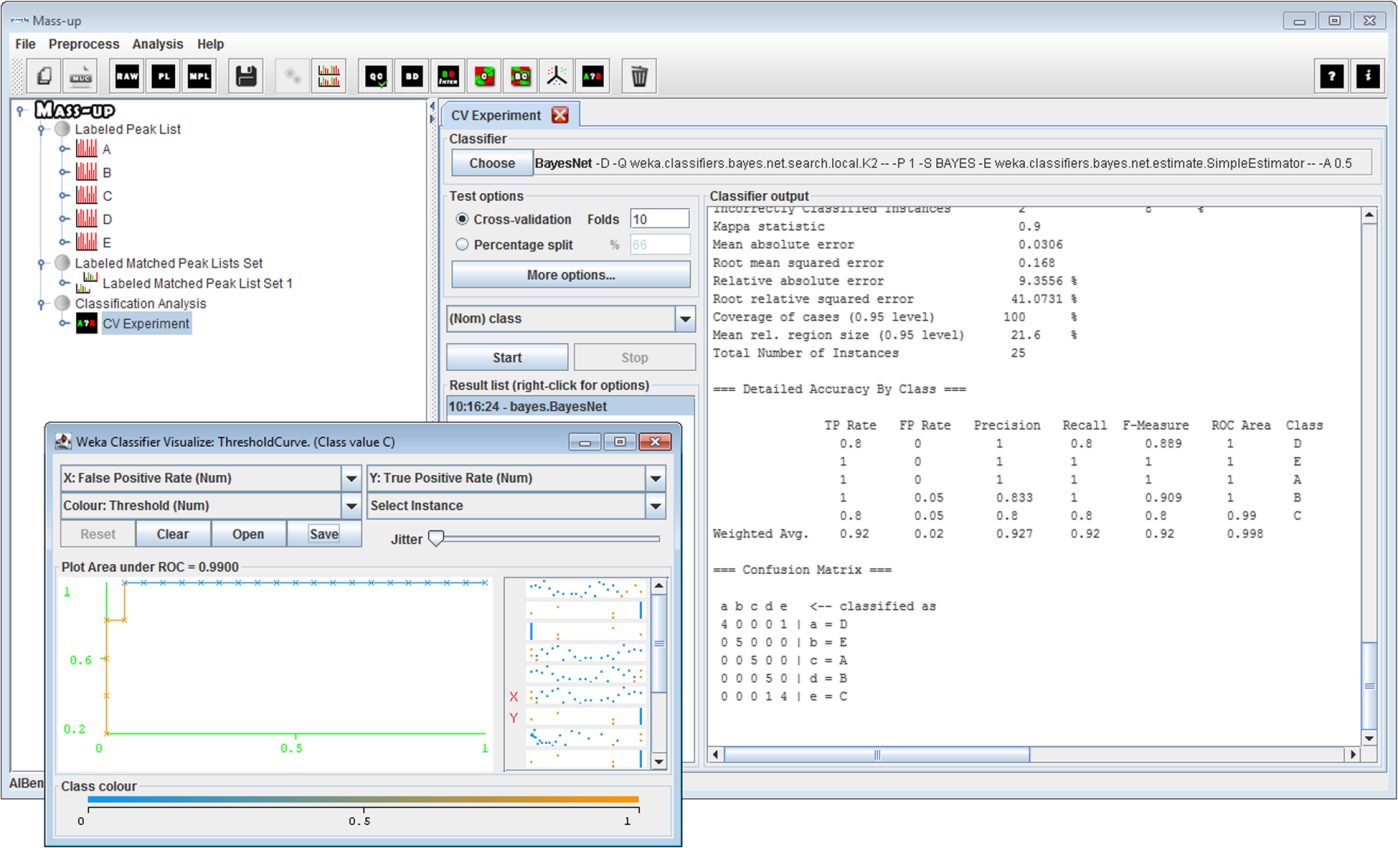
Classification analysis view. Classification analysis view presenting the result of executing a Bayes Net classifier using a 10-fold cross validation scheme. The resulting confusion matrix is presented along with several statistical measurements. ROC curve corresponding to condition C of the Wine dataset is also showed

Classification analyses are performed in the classification view (shown in Fig. 5), which was adapted from the Weka software. Through this view, the user can select a classifier and a validation scheme (i.e. cross-validation or percentage split) to perform an evaluation. As shown, the results report includes several global and per-class statistics, as well as the resulting confusion matrix. Using these operations, users can assess whether the data being analyzed is suitable for classification, as well as determine which classification algorithm is best.

### Performance notes

Although the performance is very dependent on the number of samples and the computer being used, some tests has been carried out in order to provide some performance guidelines. We have created a test dataset of 490 samples based on the Wine dataset, and then, we have executed the most common workflow of Mass-Up under an Intel Core i5 M520 with 8GB of RAM and Kubuntu 13.10 as OS. It is important to note that the size of this test dataset clearly exceeds the common size of a dataset in a MALDI-TOF MS experiment, which usually are no longer than 200 samples.

It took about 90 s to load 490 raw samples and about 200 s to fully preprocess them. Once the data is preprocessd and prior to perform any analysis, we must apply the *Match Peaks* operation, which could be executed in less than 30 s using the MALDIquant algorithm and in less than 3 s using the *Forward* algorithm. Most of the analyses (quality control, PCA, classification and intra-label analyses) could be executed in less than 5 s, while clustering, biclustering and inter-label analysis took more time. On one hand, clustering analysis took less than 20 s and the biclustering execution time depends on the algorithm selected (less than 20 s for Bibit and about 15 min for Bimax). On the other hand, inter-label biomarker discovery based on 10000 randomizations took about 8 min.

## Conclusions

In this paper we have presented Mass-Up, a new software for the analysis of MALDI data. This is an application that covers the whole process of MALDI data analysis, from data preprocessing to complex data analyses.

Mass-Up incorporates the most common analyses, aside from protein identification and focusing in biomarker discovery, such as statistical tests-based biomarker discovery, clustering, PCA, and classification. In addition, other less common analyses such as quality control and biclustering are also included. Therefore, Mass-Up provides users with a wide range of tools to analyze and explore their MALDI data.

Unlike other MS tools, Mass-Up provides a friendly graphical user interface designed to avoid the need for a bioinformatics expert to use it. The tutorial and examples included in Mass-Up tool and in the project homepage will guide users through the different operations included, making it use suitable for any user.

Finally, Mass-Up is open to further extension, such as including new operations or improving the available ones.

## Availability and requirements

The Mass-Up software is freely available from the project homepage on http://sing.ei.uvigo.es/mass-up. Additionally, source code can be downloaded from https://sourceforge.net/projects/mass-up/.

**Project name:** Mass-Up.

**Project home page:**http://sing.ei.uvigo.es/mass-up

**Operating system:** Platform independent, packaged for Windows and Linux.

**Programming language:** Java version 7.

**Other requirements:** Mass-Up has no other requirements since distrubitions are self-contained.

**License**: Version 3 of the GNU General Public License (GPLv3).

## Additional file

Additional file 1: Table S1. Detailed list of the source and version of the algorithms and libraries used in Mass-Up. (DOCX 15 kb)
