# Supplementary material for: Mass-Up: an all-in-one open software application for MALDI-TOF mass spectrometry knowledge discovery
Source: BMC Bioinformatics. 2015 Oct 5;16:318. doi: 10.1186/s12859-015-0752-4 (PMC4595311; doi:10.1186/s12859-015-0752-4)
Supplement: Additional file 1: Table S1. — Detailed list of the source and version of the algorithms and libraries used in Mass-Up. (DOCX 15 kb) [file 12859_2015_752_MOESM1_ESM.docx]

**Additional file 1**

Table S1: Algorithms and libraries used in Mass-Up.

| Task | Subtask | Algorithm | Library/Reference |
| --- | --- | --- | --- |
| Load data | Load mzXML |  | jmzReader 1.2.0 |
|  | Load mzML |  | jmzReader 1.2.0 |
|  | Load CSV |  | Own implementation |
| Preprocessing | Smoothing | Moving average window | MALDIquant 1.8 |
|  |  | Savitzky Golay | MALDIquant 1.8 |
|  | Baseline correction | SNIP | MALDIquant 1.8 |
|  |  | Convex Hull | MALDIquant 1.8 |
|  |  | Top Hat | MALDIquant 1.8 |
|  |  | Median | MALDIquant 1.8 |
|  | Normalization | Total Ion Current (TIC) | MALDIquant 1.8 |
|  |  | Probabilistic Quotient Normalization (PQN) | MALDIquant 1.8 |
|  |  | Median | MALDIquant 1.8 |
|  | Peak detection | Continuous Wavelet Transform (CWT) | MassSpecWavelet 2.12 |
|  |  | Signal-to-noise threshold | MALDIquant 1.8 |
|  | Peak matching | MALDIquant binPeaks function | MALDIquant 1.8 |
| Quality Control |  | Forward algorithm | Own implementation |
|  |  | Box-and-whiskers plotting | JFreeChart 1.0.13 |
| Biomarker Discovery | Intra-label |  | Own implementation |
|  | Inter-label | Chi-square test | Apache Commons Math 3.0 |
|  |  | Fisher exact test | Own implementation |
|  |  | Randomization test | Own implementation  Apache Commons Math 3.0 |
|  |  | Yates’ chi-squared test | Own implementation |
|  |  | Benjamini & Hochberg FDR correction | Own implementation |
| Clustering | Clustering computation | Agglomerative Hierarchical Clustering | Own implementation |
|  | Clustering visualization |  | JTreeView |
| Biclustering |  | Bimax | BicAT (Biclustering Analysis Toolbox) |
|  |  | BiBit | http://www.upo.es/eps/bigs/BiBit_algorithm.html |
| Classification Analysis | Classifiers | Weka | Weka 3.6 |
| Principal Component Analysis | PCA computation | Weka | Weka 3.6 |
|  | PCA visualization |  | Jzy3d 0.9 |
